# Usability and preliminary effectiveness of an app-based physical activity and education program for people with hip or knee osteoarthritis – a pilot randomized controlled trial

**DOI:** 10.1186/s13075-024-03291-z

**Published:** 2024-04-10

**Authors:** F. Weber, C. Kloek, S. Stuhrmann, Y. Blum, C. Grüneberg, C. Veenhof

**Affiliations:** 1 Department of Applied Health Sciences, Division of Physiotherapy, Hochschule für Gesundheit, University of Applied Health Sciences, Gesundheitscampus 6-8, Bochum, 44801 Germany; 2 Department of Rehabilitation, Physiotherapy Science & Sports, UMC Utrecht Brain Center, University Medical Center Utrecht, Utrecht University, Utrecht, The Netherlands; 3 Research Group Innovation of Human Movement Care, Knowledge Centre Healthy and Sustainable Living, HU University of Applied Sciences, Utrecht, The Netherlands

**Keywords:** Osteoarthritis, Physical activity, Education, Exercise, Digital health, Pilot study

## Abstract

**Background:**

Hip and knee osteoarthritis (OA) are highly prevalent worldwide. The guidelines recommend physical activity and education as the core treatments for osteoarthritis. Digital health has the potential to engage people in physical activity and disease management. Therefore, we conducted a pilot trial to assess the usability and preliminary effectiveness of an app-based physical activity and education program (*Join2Move*) compared to usual care for people with hip and/or knee OA in Germany.

**Methods:**

A randomized controlled pilot study was conducted. Individuals with diagnosed or self-reported knee and hip OA were included. Allocation to the intervention or control group was randomized. The intervention group received the *Join2Move* program. The *Join2Move* program was previously developed as a website and evaluated in the Netherlands. For the current study, the program was translated and adapted to the German context and adjusted from a website to an app. The control group received usual care. The primary outcomes were usability and preliminary effectiveness (pain and physical functioning). Measurements were taken at baseline and at twelve weeks. The data analysis was performed using SPSS (IBM SPSS Statistics 29.0).

**Results:**

Sixty participants, with a mean age of 61.9 (SD ± 7.2) years, were allocated to the intervention (*n* = 32) or the control group (*n* = 28) and included in the analysis. The majority of participants had knee OA (68%), and 12% had hip and knee OA. The dropout rate was *n* = 11 (18%). No adverse events were reported. Usability was rated as acceptable (mean System Usability Scale = 71.3/100) with a wide range (32.5 to 100). Statistically significant between-group differences were found only for pain (mean difference 8.52 (95% CI 1.01 to 16.04), *p* = 0.027).

**Conclusions:**

*Join2Move* demonstrated acceptable usability. The preliminary results of the pilot trial indicate the potential of a stand-alone app for the treatment of patients with hip or knee OA. However, the acceptable usability of *Join2Move* limits its recommendation for everyone. There appears to be room for improvement in app usability and in identifying patients for whom the app is suitable and the right time to use a stand-alone app.

**Trial registration:**

German Clinical Trials Register DRKS00027164.

**Supplementary Information:**

The online version contains supplementary material available at 10.1186/s13075-024-03291-z.

## Background

Osteoarthritis (OA) is the leading joint disease worldwide and affects approximately half of the population over the age of 65 [1, 2]. In the Western world, it is one of the most common causes of pain, functional impairment and disability in adults and affects quality of life [3]. OA most commonly affects the hip and knee joints [4, 5]. For instance, obesity and physical inactivity are known modifiable risk factors for the development of OA [6]. In Germany, as in other Western countries, the number of people with OA is further increasing [4, 5]. Therefore, there is a need for accessible and effective support for this group.

The guidelines recommend physical activity and education as the cornerstones in the care of people with hip and knee OA [7–9]. Furthermore, there is a consensus in the literature that conservative treatments are preferable to surgery for the management of OA [7–9]. General practitioners (GPs) are regularly the first and main points of contact for people with OA. Consequently, GPs would be the ideal person for the first step of the stepped care strategy to promote physical activity and exercise [10]. However, the ability of GPs to promote physical activity is limited by time constraints and a lack of standard protocols. Moreover, people in the early stage of OA often do not receive help elsewhere. Thus, the vast majority of people diagnosed with hip or knee OA are not adequately treated [11–13]. Further tools to support patient self-care are therefore needed [14]. In addition to the increasing need for chronic care, the decreasing capacity of the health care system poses an additional challenge for an aging society, leading to a shift toward home care and an increasing emphasis on patient self-management [15]. In particular, the management of patients with early-stage OA, which also refers to the stepped care strategy, may benefit from digital treatment, such as an app-based intervention [9, 10]. Mobile health apps offer an opportunity to reduce this gap by providing both exercise and physical activity support, specific and tailored information and education [16, 17]. A recent systematic review and meta-analysis by Xie et al. (2021) on web-based interventions in patients with knee OA provided evidence that such interventions can improve pain and physical functioning in patients with OA [18].

For instance, one existing web-based program called *Join2Move* was developed in the Netherlands by Bossen et al. (2013). This web-based program has been previously researched and found to be effective [19, 20]. As digital health interventions need to be adapted to specific contexts (e.g., the German health care context) and can quickly become outdated, we translated, adapted and upgraded the web-based *Join2Move* program into a German app.

Therefore, the aim of this randomized controlled pilot study was to assess the usability of the app-based *Join2Move* program for people with hip and/or knee OA. Furthermore, the preliminary effectiveness of the program on pain and physical functioning over twelve weeks was investigated.

## Methods

### Study design

A two-armed, assessor-blinded, randomized controlled pilot study was conducted, focusing on the usability and preliminary effectiveness of the app-based *Join2Move* program. Reporting was based on the *Consolidated Standards for Reporting Trials (CONSORT) extension for randomized pilot and feasibility trials* for transparent reporting [21]. The study was registered in the German Clinical Trial Register (DRKS: DRKS00027164). The ethics committee of the University of Applied Health Sciences Bochum approved the study (210828_Grüneberg, 10.11.2021). Informed consent was obtained from each participant before enrollment.

### Participants, recruitment and procedures

Individuals with diagnosed or self-reported knee OA older than 38 years and hip OA older than 50 years were included in the study. Patients with self-reported OA had to meet the *American College of Rheumatology* criteria for inclusion in the study [22, 23]. The criteria were verified by clinical examination and interviews with study staff.

Potential participants were recruited through newspaper announcements and a press release. In a subsequent telephone call, they were assessed for potential eligibility and, if deemed eligible, were invited to the University of Applied Health Sciences Bochum, where the baseline and final measurements were administered. The twelve-week intervention was conducted at the participants’ homes or at the location of their choice.

People were excluded if they (1) did not have internet access at home, (2) did not own a smartphone or tablet, or (3) could not read or understand the German language. People were also excluded if they (4) were on a waiting list for joint replacement surgery for their affected joint or had already undergone joint replacement surgery for their affected joint or (5) had contraindications (e.g., loss of consciousness or cardiovascular disease) to physical activity without medical supervision. If a clear decision for inclusion could not be made, a physician’s consent was needed. Furthermore, individuals were excluded if they (6) had received physiotherapy and/or specialist treatment for OA in the previous six months. Eligible persons were asked to bring the completed questionnaires to their first appointment at the University of Applied Health Sciences Bochum. These were mailed to them together with the informed consent form and study information. At the on-site appointment, three examiners (with a degree in physiotherapy) who had previously received adequate training in all steps of the measurement (4 h training) performed the initial measurements.

Participants in the intervention group received a handout from the study staff with information on how to install the app and instructions on how to get started. In addition, they were asked not to receive any physiotherapy for their affected joint during the study period. The control group was free to receive usual care, e.g., physiotherapy, which is covered by statutory health insurance funds in Germany.

Six weeks after their first measurement appointment, all study participants were contacted by email to complete a short self-developed online questionnaire as a quick reminder of their participation in the study. The participants were asked about any adverse events and complaints they had experienced during the previous 6 weeks.

Before the final measurements were taken at twelve weeks, all the necessary questionnaires were sent to the subjects. Blinded examiners performed the follow-up measurements. Participants were able to contact study staff by email or telephone to report any adverse events or questions.

### Randomization

Immediately after the clinical examinations, participants were randomized into the intervention or control group. Assignment was based on computer-generated randomization performed by staff who were not involved in the measurements. The study administrator personally informed the participants of their assignment. The number assigned to the subjects was saved in a password-protected code list. The study administrator did not personally perform any of the follow-up assessments. After the follow-up measurements, participants in the control group were given the opportunity to download and use the *Join2Move* app free of charge.

### Outcome measures

To characterize the population, a questionnaire with general demographic data (age, sex, height, weight) was completed. In addition, data were collected on education level, occupation and information on the disease, e.g., affected joint, duration of OA, comorbidities, use of assistive devices, symptoms and physiotherapeutic care. Furthermore, health literacy was assessed with the *European Health Literacy Questionnaire* (HLS-EU-Q16), while the *eHealth Literacy Scale* (eHEALS) was used to determine digital health literacy [24, 25]. On the HLS-EU-Q16, scores between 0–8 are considered as inadequate, 9–12 as problematic and 13–16 as adequate [24]. Several studies have described high levels of digital health literacy, with a score of 26 (scale 8–40) on the eHEALS [26].

#### Primary outcome measures

##### Usability

The German version of the *System Usability Scale* (SUS) (0–100) was used to assess usability [27]. A score of < 50 was considered unacceptable, 50–70 was considered marginal, and > 70 was considered acceptable (> 85 = excellent) [28].

##### Pain and physical functioning

To examine pain and physical functioning in daily living, the *Hip Disability and Osteoarthritis Outcome Score* (HOOS) pain and functioning in daily living subscales for subjects with hip OA [29] and the *Knee Injury and Osteoarthritis Outcome Score* (KOOS) pain and functioning in daily living subscales for subjects with knee OA [30] (0–100) were used.

#### Secondary outcome measures

##### Usability

We used the Thinking Aloud procedure to consider how end-users interact with the intervention. Therefore, five randomly selected participants were asked to accomplish the selected tasks within the app while expressing their thoughts aloud. The feedback was recorded with the help of audio recordings, and the time was stopped for each task. This sample size is sufficient to log 85% of the usability problems [31].

##### Satisfaction

Patient satisfaction with the app-based care was assessed with the modified ZUF-8 [32]. The questionnaire obtains values ranging from eight to 32. Low values are associated with poor patient satisfaction, and high values are associated with good patient satisfaction.

The *Join2Move questionnaire* (Additional file 1) is a self-developed instrument for determining subjective usage time, user satisfaction and usability of the *Join2Move* app and its individual modules. In addition, information on any symptoms and adverse events that may have occurred was collected.

##### Strength

The strength of the knee flexors and extensors, hip flexors, extensors and abductors was measured. Hip muscle strength was measured isometrically using a handheld dynamometer (*Hoggan MicroFET 2*), with three replicates collected for each muscle group, and the results were calculated as the means [33]. Isokinetic strength measurements of the knee flexors and extensors were performed using a *Biodex System 4*. A five-minute nonspecific warm-up was performed on the bicycle ergometer prior to the measurements. The measurement started on the unaffected or less affected side with a specific movement preparation of 20 repetitions at 60°/s. The range of motion was previously set to 90° of flexion and possible extension. A trial run and the actual measurement, with four repetitions each at 60°/s, were connected. The examination was repeated on the more affected side. Subsequently, a measurement at 120°/s was performed according to the same protocol [34].

The *30 Second Sit to Stand Test* assesses individuals’ functional leg strength and endurance. Participants were asked to sit on a chair with a seat height of 44 cm and stand up as many times as possible within 30 s without using their arms for support. The number of standing repetitions was counted [35].

##### Range of motion

The range of motion in knee flexion and extension and in hip flexion, extension, abduction, internal rotation and external rotation was assessed using an analog goniometer [36].

##### Physical activity

The *International Physical Activity Questionnaire* (IPAQ) measures subjective levels of physical activity and categorizes individuals into light (walking), moderate and vigorous activity over the past seven days. In addition, sedentary time is measured [37].

##### Self-management

The German version of the *Patient Activation Measure* (0–100) is a well-established, validated instrument for measuring active patient participation and the level of self-management [38].

### Intervention

The app-based *Join2Move* program includes a twelve-week exercise, physical activity and education program for knee and hip OA patients. It is based on the Dutch *Join2Move* program developed by Bossen and colleagues and consists of three modules: (1) graded physical activity, (2) exercise and (3) education [20]. In contrast to the Dutch version, the program was delivered via an app and not a website. Furthermore, the type of exercise was changed since we included the evidence-based *NEuroMuscular EXercise* (*NEMEX*) program and new educational content, which was based on the Dutch *e-Exercise* program for patients with hip or knee OA developed by Kloek and colleagues [39].

**Fig. 1:**
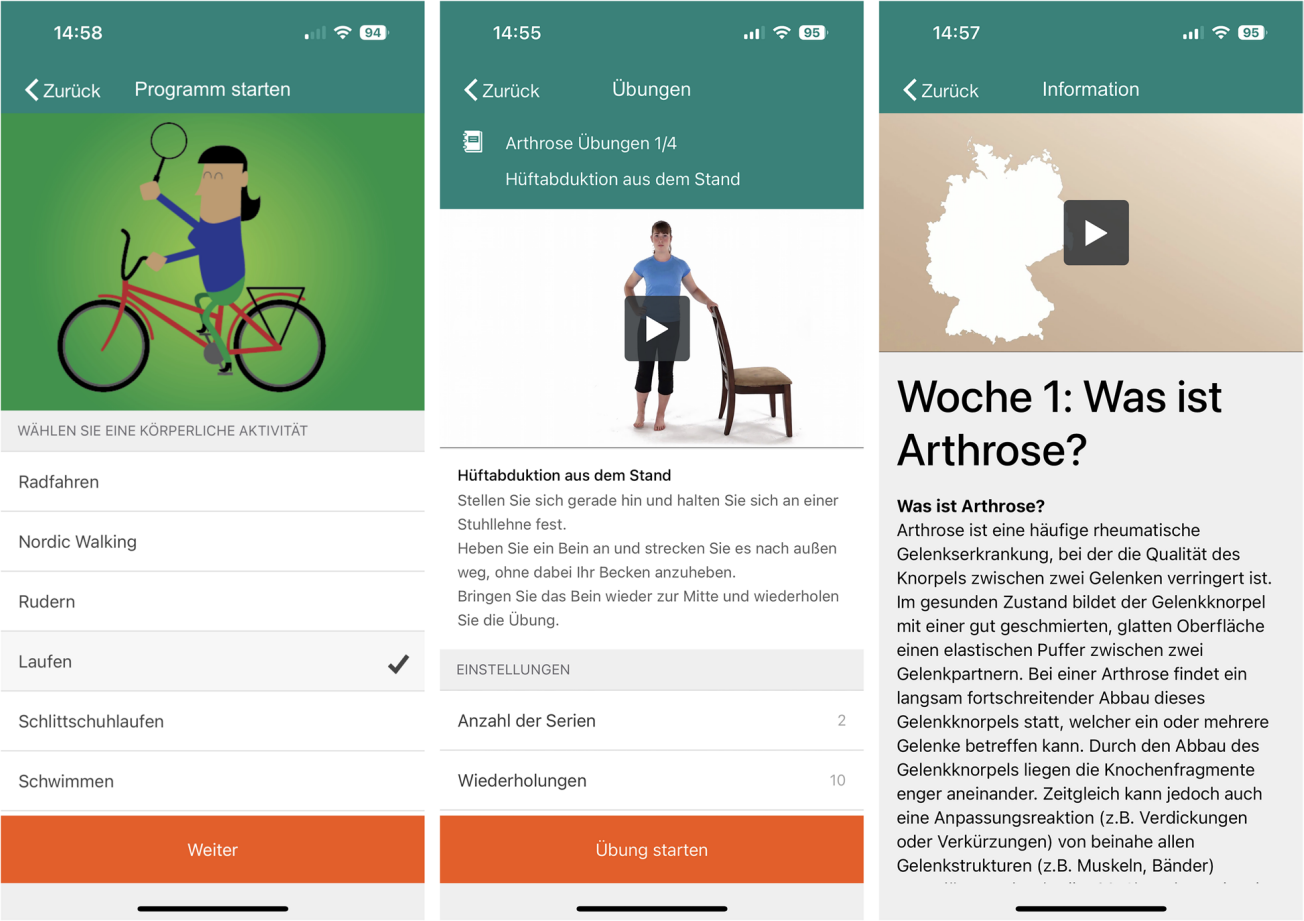
Screenshots of the German Join2Move app showing the module physical activity (left) (choosing a physical activity such as running or swimming and start the program), exercise (center) (one exercise along with the description of the exercise and customized setting) and education (right) (educational unit on the topic "What is osteoarthritis?". Pressing the button would start an explanatory video on this topic)

(1) The physical activity module consists of a baseline measurement taken during the first week. First, participants choose from a range of activities (e.g., swimming or cycling) (Fig. 1). The participants were then asked to perform three days of activities for the week. Finally, they set a short-term goal to achieve at the end of the program. The duration of the chosen physical activity is gradually increased over the next twelve weeks (according to the principle of graded activity) until the individual short-term goal is reached [40, 41]. (2) The exercise module provides participants with two or three video-based exercises three days per week. The number of repetitions is gradually increased. The exercise module based on the *NEMEX* program focuses on four domains: core stability/postural function, postural orientation, lower extremity muscle strength and functional exercises (Fig. 1) [42]. An overview of the integrated exercise program can be found in Additional file 2. *NEMEX* was shown to reduce pain and increase physical activity in patients with knee or hip OA [43, 44]. (3) In addition, each week, participants received a new video or text in the education module with small assignments at the end of each module (e.g., “Symptoms of OA”; “Self-management and OA”; “Exercise despite pain?”) (Fig. 1). These education modules were based on the Dutch *e-Exercise* program for patients with hip or knee OA [39]. Weekly reminders were sent to remind the participants of new tasks and content. Overall, the app program was translated and adapted to the German language and context.

### Sample size

The sample size for this pilot study was set at 60 subjects. A minimum of twelve to 35 subjects per group is advised for pilot studies [45, 46]. Teare et al. (2014) recommend a sample size of at least 70 to 120 to estimate the standard deviation and event rate [46]. However, smaller sample sizes are recommended for simple calculations [47], which fit our main objective of investigating the usability and preliminary effectiveness of the *Join2Move* program.

### Data analysis

The data were analyzed using SPSS (IBM SPSS Statistics 29.0). Data from participants who did not complete the study were included using the intention-to-treat (ITT) method. The Shapiro‒Wilk test was used to test the normality of the outcome data. If variables were not normally distributed, they were log-transformed. The results of the sociodemographic characteristics of the participants, health literacy and digital health literacy, app usage, handling of the app, extent of OA-specific complaints, physical activity and self-management of the participants, and usability and satisfaction were presented descriptively. Therefore, means with standard deviations (SDs); medians with interquartile ranges; variances; minimums; and maximums were calculated. Selected data (primary and secondary outcome measures) were explored for differences within and between groups using inferential statistics. The appropriate inferential statistical procedure was chosen depending on the sample size, scale and distribution of the data. Analyses of covariance (ANCOVAs) were used to calculate the preliminary effectiveness of the primary and secondary outcomes. Effect sizes and 95% confidence intervals (CIs) were calculated to measure clinical meaningfulness. Effect sizes were expressed in partial eta squared (ƞ_p_^2^), with values of 0.01, 0.06, and 0.14 representing small, medium, and large effects, respectively [48]. For statistical evaluation, the values of the more affected side or joint (self-reported) were used. If it was unknown, which side was most affected, the right side was selected for analysis. Thinking Aloud recordings were transcribed, coded, organized and evaluated in terms of protocol analysis using MAXQDA Plus 2020, Windows version 20.3.0 [49]. The time to complete the tasks is presented as the mean and range.

## Results

### Participants

**Fig. 2:**
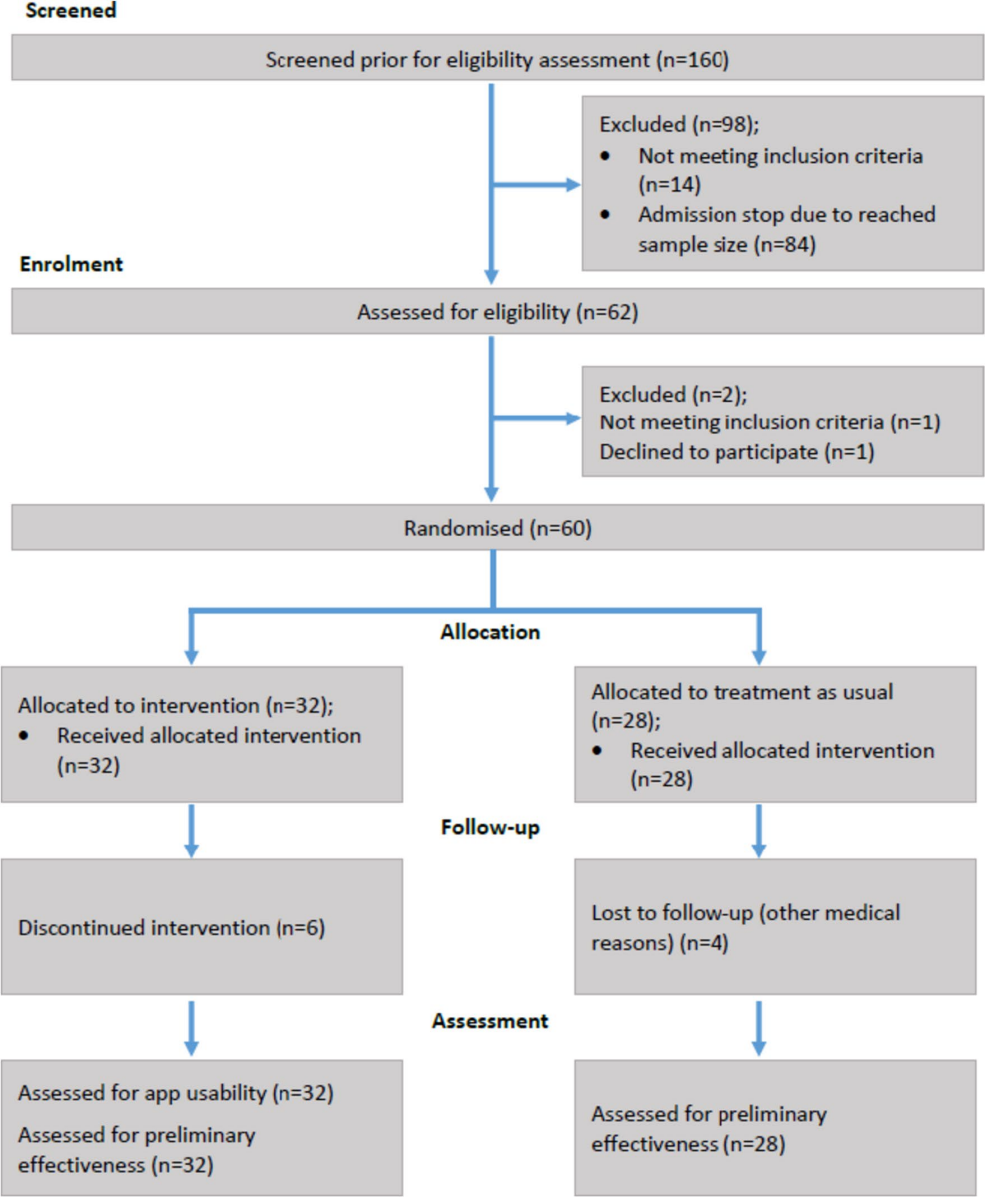
Consolidated Standards for Reporting Trials (CONSORT) flow diagram

The participant flow is illustrated in Fig. 2 following the CONSORT template [50]. Initially, 61 people with knee and/or hip OA were enrolled in the pilot study. During the baseline measurement, one person was excluded since the person could not perform isokinetic strength measurements without an increase in pain. Sixty people completed the baseline measurements and were randomized into the intervention (*n* = 32) or the control group (*n* = 28). The dropout rate was *n* = 11 (18%).

The baseline characteristics of the patients are presented in Table 1. The mean age of the participants was 62 (SD ± 7), and almost 2/3 of the participants were female. The majority of participants were affected by knee OA (68), with 12% having both knee and hip OA. Most of the participants had been affected by OA for more than ten years. The baseline characteristics of the two groups were similar for demographic, primary, and secondary outcome measures. At baseline, complete data on outcome measures were available for 100% (60/60) of the participants. At the 3-month follow-up, complete data on outcome measures were available for 81% (26/32) of the patients in the intervention group and 86% (24/28) of those in the usual care group. No serious adverse events were reported in the intervention group.

**Table 1:** Demographic and clinical characteristics

**Characteristics**	**Group**		
	**Join2Move** (*n* = 32)	**Usual care** (*n* = 28)	**Total** (*n* = 60)
**Mean age (SD), *y***	60 (6)	64 (8)	62 (7)
**Female, n (%)**	21 (66)	16 (57)	37 (62)
**Mean body mass index (SD), *kg/m***^***2***^	28 (6)	28 (5)	28 (6)
**Location of osteoarthritis, n (%)**			
Hip	9 (28)	3 (11)	12 (20)
Knee	20 (63)	21 (75)	41 (68)
Both	3 (9)	4 (14)	7 (12)
**Symptom duration, n (%)**			
< 1 *y*	1 (4)	0 (0)	1 (2)
1–5 *y*	6 (23)	9 (38)	15 (30)
5–10 *y*	8 (31)	8 (33)	16 (32)
> 10 *y*	11 (42)	7 (29)	18 (36)
**Level of education, n (%)**			
Low	0 (0)	2 (7)	2 (3)
Middle	15 (48)	11 (39)	26 (44)
High	16 (52)	15 (54)	31 (53)
**Employment status, n (%)**			
Currently employed	17 (53)	12 (43)	29 (48)
Retired	11 (34)	15 (54)	26 (43)
Unemployed/student	2 (6)	1 (4)	3 (5)
Homemaker	2 (6)	0 (0)	2 (3)
**Comorbidities, n (%)**			
Cardiovascular	11 (34)	14 (50)	25 (42)
Neurological	0 (0)	1 (4)	1 (2)
Musculoskeletal	2 (6)	3 (11)	5 (8)
Respiratory	0 (0)	1 (4)	1 (2)
Metabolic	8 (25)	4 (14)	12 (20)
Others	2 (6)	2 (7)	4 (7)
**Subjective limitations, n (%)**			
None	0 (0)	0 (0)	0 (0)
Low	14 (44)	10 (36)	24 (40)
Fair	15 (47)	16 (57)	31 (52)
Strong	3 (9)	2 (8)	5 (8)
**Mean health literacy (SD)**			
**HLS-EU-Q16 (0–16)**	13 (3)	13 (3)	13 (3)
**eHEALS (8–40)**	30 (6)	29 (4)	29 (5)

Continuous variables were expressed as the mean and standard deviation (SD) and categorical variables as number (n) and percentage (%)

*n* number, *SD* Standard deviation, *HLS-EU-Q16* European Health Literacy Questionnaire (0–16), *eHEALS* eHealth Literacy Scale (8–40)

### Usability and satisfaction

The overall usability assessed with the SUS revealed a mean score of 71.3/100 (SD 18.2), with a range from 32.5 to 100. The mean patient satisfaction score was 23.8/32 (SD 4.3), with a range from 16 to 32. Table 2 shows the results of the usability and usage of the *Join2Move* app in general and of the specific modules (exercises, physical activity and education). Most of the participants used the app more than three times a week (16 (61.5%)). The majority of participants found it very easy to find the different modules in the app. The usefulness of the different modules was rated as “valuable” by most of the participants.

**Table 2:** Outcomes of the set of questions related to Join2Move (*n* = 26)

**Questionnaire related to the usability of the *Join2Move* app**	
How often did you use the app in the last 12 weeks?	
≥ *3 times/week*	16 (61.5)
< *3 times/week*	10 (38.5)
*Not at all*	0 (0)
***Module: exercises***	
How often did you perform the exercises via the app?	
≥ *3 times/week*	14 (53.9)
< *3 times/week*	11 (42.3)
*Not at all*	1 (3.8)
How difficult/easy was it to find the exercise module?	
*Very difficult*	0 (0)
*Difficult*	5 (19.2)
*Easy*	6 (23.1)
*Very easy*	15 (57.7)
How would you rate the usefulness/value of the exercises?	
*Not valuable at all*	2 (7.7)
*Not valuable*	2 (7.7)
*Valuable*	18 (69.2)
*Very valuable*	4 (15.4)
***Module: physical activity***	
How often did you perform the chosen physical activity (e.g. walking, cycling, swimming …) in the app?	
≥ *3 times/week*	14 (53.9)
< *3 times/week*	9 (34.6)
*Not at all*	3 (11.5)
How difficult/easy was it to find the physical activity module?	
*Very difficult*	0 (0)
*Difficult*	2 (7.7)
*Easy*	8 (30.8)
*Very easy*	16 (61.5)
How would you rate the usefulness/value of the physical activity module?	
*Not valuable at all*	2 (7.7)
*Not valuable*	5 (19.2)
*Valuable*	14 (53.9)
*Very valuable*	5 (19.2)
***Module: education***	
How often did you use the educational material (e.g. videos) in the app?	
≥ *3 times/week*	5 (19.2)
< *3 times/week*	18 (69.2)
*Not at all*	3 (11.5)
How difficult/easy was it to find the education module?	
*Very difficult*	0 (0)
*Difficult*	0 (0)
*Easy*	10 (38.5)
*Very easy*	16 (61.5)
How would you rate the usefulness/value of the education module?	
*Not valuable at all*	1 (3.8)
*Not valuable*	1 (3.8)
*Valuable*	20 (76.9)
*Very valuable*	4 (15.4)
**Usability and satisfaction with the Join2Move app**	
	**M (SD)**
How would you rate the app usability in general? (0–10)	6.0 (2.5)
How satisfied are you in general with the app? (0–10)	6.0 (2.6)

#### Thinking Aloud approach

Table 3 summarizes the ten tasks and the average time, as well as the range of time that the participants needed to complete the tasks. In general, participants found it easy and simple to navigate through the app. After completing the Thinking Aloud approach, four of the participants concluded that they truly liked the app and thought, “this app is suitable for me”. In addition, they mentioned that the home screen provided a useful overview of the content of the app and was a good starting point for everyone, as emphasized in the quotation of one participant: “You need to navigate back to the home screen and then click on “About this app”. Already found it, that was easy.” However, there were some usability issues. For example, the third task was a challenge for all participants, as they had to find the settings to which the reminders were linked. Furthermore, the fourth task was quite challenging for all participants. They found it difficult to distinguish between exercises, training programs and physical activity. For instance, one participant looked for exercises when trying to start the training program. In general, tasks 8, 9 and 10 were easy to complete, but participants were sometimes confused about the word ‘information’. They expected some general information about the app and not information on OA-related issues.

**Table 3:** Thinking Aloud test results among participants (*n* = 5)

No	Tasks	Average time (range) in sec
**Navigation**		
1	Navigate to the home screen	6 (3 to 10)
2	Navigate to “About this app”	8 (3 to 11)
3	Activate a reminder for performing the exercises and physical activities. Make sure that you also receive a notification about it	92 (30 to 280)
**Performance**		
4	Create a training program and set a goal for a physical activity	109 (37 to 154)
5	Set the amount of series and repetitions of the first exercise	50 (16 to 88)
6	Set a reminder to perform the exercises and determine on which days you would like to perform them	28 (18 to 35)
7	Watch the video of the first exercise	22 (10 to 47)
**Search for/collect information**		
8	Search for information regarding a healthy body weight and osteoarthritis	27 (13 to 58)
9	Search for information on the influence of stress on osteoarthritis	13 (4 to 25)
10	Start the information video for week 2	51 (10 to 103)

### Preliminary effectiveness

#### Pain and physical functioning

There were statistically significant and clinically important between-group differences in the primary outcome pain at t1 (adjusted mean difference of 8.52; 95% CI 1.01 to 16.04; *p* = 0.027). For physical functioning, there was no statistically significant between-group difference at t1 (adjusted mean difference of 5.37; 95% CI -1.57 to 13.03; *p* = 0.121). There were significant within-group differences in pain in the intervention group (-5.81; 95% CI -11.34 to -0.28; *p* = 0.020); however, there were no differences in physical functioning (-3.66; 95% CI -9.49 to 2.17; *p* = 0.105). For the control group, there were no significant within-group differences in pain (0.40; 95% CI -6.01 to 6.81; *p* = 0.449) or physical functioning (0.66; 95% CI -5.55 to 6.88; *p* = 0.414).

#### Secondary outcomes

The only significant between-group differences at t1 were found in an isokinetic strength measurement (flexion 60° total work) and in the International Physical Activity Questionnaire (IPAQ). However, the isokinetic strength (flexion 60° total work) decreased from t0 to t1, and the between-group difference in the IPAQ score was in favor of the control group. For all the other secondary outcomes, there were no statistically significant between-group differences with low to moderate effect sizes (Table 4).

**Table 4:** Primary and secondary outcome measures used to assess preliminary effectiveness from baseline (t0) to follow-up (t1) (12 weeks)

**Outcome**	**Baseline (t0)**		**Week 12 (t1)**		**Mean Difference (95% CI for Difference)**	**ES (ƞ**_**p**_^**2**^**)**	***p* value**
	**Intervention (*n* = 32)**	**Control (*n* = 28)**	**Intervention (*n* = 32)**	**Control (*n* = 28)**			
**Primary**							
HOOS/KOOS pain^a^	62.5 (16.8)	55.9 (23.7)	68.3 (16.1)	55.5 (22.5)	8.52 (1.01 to 16.04)	0.08	0.027^*^
HOOS/KOOS ADL^b^	68.1 (20.2)	64.3 (23.0)	71.8 (14.3)	63.7 (23.9)	5.73 (-1.57 to 13.03)	0.04	0.121
**Secondary**							
HOOS, KOOS total^c^	52.7 (18.0)	50.3 (20.5)	56.0 (13.0)	50.0 (21.4)	4.72 (-1.60 to 11.04)	0.04	0.140
HOOS/KOOS symptoms^c^	58.7 (20.1)	54.2 (21.5)	59.3 (17.1)	55.1 (22.1)	0.91 (-5.75 to 7.58)	0.00	0.785
HOOS/KOOS sport^c^	39.7 (25.6)	41.6 (29.2)	41.7 (17.6)	35.3 (28.5)	7.16 (-3.55 to 17.86)	0.03	0.186
HOOS/KOOS QoL^c^	34.9 (19.0)	35.5 (20.5)	40.4 (13.0)	38.9 (23.4)	1.83 (-6.22 to 9.87)	0.00	0.651
***ROM Knee (in °)***	***n* = 22**	***n* = 23**	***n* = 22**	***n* = 23**			
Flexion	125.0 (13.4)	121.8 (13.7)	126.8 (15.4)	124.3 (11.5)	0.59 (-6.09 to 7.26)	0.00	0.860
Extension	0.3 (5.7)	-2.8 (8.0)	-3.5 (5.5)	-2.7 (6.7)	-1.69 (-5.35 to 1.96)	0.02	0.355
***ROM Hip (in °)***	***n* = 10**	***n* = 5**	***n* = 10**	***n* = 5**			
Flexion	101.2 (17.1)	109.0 (17.5)	115.5 (9.0)	118.0 (5.7)	-0.92 (-10.22 to 8.39)	0.00	0.834
Extension	16.5 (6.7)	13.0 (7.6)	17.1 (6.2)	16.6 (6.5)	-1.07 (-8.11 to 5.96)	0.01	0.745
External Rotation	28.8 (10.4)	24.0 (19.5)	32.7 (9.7)	24.2 (15.3)	5.34 (-3.92 to 14.59)	0.12	0.233
Internal Rotation	23.0 (13.8)	28.0 (10.4)	24.7 (11.4)	31.2 (18.4)	-4.35 (-20.59 to 11.89)	0.03	0.570
Abduction	33.0 (11.6)	29.0 (7.4)	32.9 (3.3)	29.4 (11.5)	2.76 (-5.67 to 11.19)	0.04	0.490
***Isokinetic Strength Knee***	***n* = 32**	***n* = 28**	***n* = 32**	***n* = 28**			
*Extension (60°/sec)*							
Peak Torque (*Nm*)	100.6 (37.6)	100.6 (40.2)	96.4 (26.9)	98.8 (34.6)	-2.49 (-13.58 to 8.60)	0.00	0.655
Total Work (*J*)	316.7 (114.3)	305.2 (137.6)	303.1 (91.2)	285.4 (105.8)	11.04 (-23.45 to 45.54)	0.01	0.524
Avg. Power (*W*)	56.8 (20.9)	57.8 (28.1)	56.2 (17.2)	55.9 (23.0)	0.90 (-6.30 to 8.09)	0.00	0.803
*Extension (120°/sec)*							
Peak Torque (*Nm*)	80.0 (28.0)	79.0 (30.8)	76.5 (20.2)	74.4 (26.8)	1.47 (-6.51 to 9.46)	0.00	0.713
Total Work (*J*)	255.8 (87.1)	243.5 (109.4)	221.5 (73.0)	203.4 (82.8)	11.08 (-17.27 to 39.43)	0.01	0.437
Avg. Power (*W*)	79.0 (29.9)	80.4 (40.6)	68.6 (24.5)	64.5 (30.5)	4.92 (-5.18 to 15.02)	0.02	0.333
*Flexion (60°/sec)*							
Peak Torque (*Nm*)	77.9 (29.1)	69.6 (29.7)	75.7 (22.4)	69.4 (19.8)	1.85 (-5.71 to 9.41)	0.00	0.625
Total Work (*J*)	303.5 (119.5)	262.9 (126.0)	289.0 (91.8)	237.5 (79.1)	29.70 (0.21 to 59.19)	0.07	0.048^*^
Avg. Power (*W*)	48.6 (18.8)	44.7 (21.4)	49.8 (16.1)	43.7 (16.4)	3.79 (-1.86 to 9.44)	0.03	0.184
*Flexion (120°/sec)*							
Peak Torque (*Nm*)	71.0 (25.5)	65.3 (25.8)	64.4 (19.7)	58.8 (18.8)	3.10 (-3.52 to 9.71)	0.02	0.352
Total Work (*J*)	280.5 (99.3)	240.3 (115.1)	219.8 (77.2)	183.5 (69.8)	17.26 (-11.38 to 45.90)	0.03	0.232
Avg. Power (*W*)	76.2 (31.3)	69.0 (30.9)	62.9 (24.8)	53.4 (23.0)	5.86 (-3.54 to 15.27)	0.03	0.217
***Isometric Strength Hip (kg)***							
Flexion	14.1 (4.3)	15.2 (5.4)	20.4 (5.1)	19.5 (4.7)	1.26 (-1.20 to 3.71)	0.02	0.309
Extension	18.3 (6.9)	20.9 (6.7)	22.1 (7.0)	23.0 (7.2)	0.22 (-3.16 to 3.59)	0.00	0.898
Abduction	15.7 (4.8)	16.8 (5.5)	17.5 (5.2)	19.1 (5.7)	-1.05 (-3.54 to 1.45)	0.01	0.404
***30 Seconds Sit to Stand Test*** (*Rep*)	14.5 (3.9)	15.1 (4.8)	16.6 (3.5)	17.5 (6.4)	-0.38 (-2.15 to 1.40)	0.00	0.673
***IPAQ*** (*METmin/week*)	2941.6 (3049.1)	3123.3 (2468.4)	3135.3 (1836.4)	4517.2 (3398.6)	-1291.93 (-2491.99 to -91.88)	0.08	0.035^*^
***PAM-13*** (0–100)	66.9 (11.5)	66.9 (10.7)	70.3 (10.6)	65.7 (11.0)	4.67 (-0.19 to 9.53)	0.06	0.059

*CI* confidence interval, *ES* effect size (partial eta squared), *ADL* activity of daily living, *HOOS* hip disability and osteoarthritis outcome score, *KOOS* knee injury and osteoarthritis outcome score, *NRS* numerical rating scale, *QoL* Quality of Life, *IPAQ* International Physical Activity Questionnaire, *PAM-13* Patient Activation Measure

*Values in parentheses are SDs

^a^Ranges from 0 to 100; lower scores indicate more pain

^b^Ranges from 0 to 100; lower scores indicate worse function

^c^Ranges from 0 to 100; lower scores indicate more knee problems

## Discussion

The aim of this pilot randomized controlled trial was to investigate the usability and preliminary effectiveness of the app-based *Join2Move* program in patients with knee and/or hip OA. The expected adequate usability of the app was confirmed by the results, which revealed acceptable usability of the SUS (M (SD) = 71.3/100 (18.2)) and was supported by the findings of the Thinking Aloud approach. A significant and clinically important reduction in pain was found in favor of the intervention group. However, there were no significant effects on physical functioning.

Although the usability scores were acceptable, the range of the scores was quite large, suggesting that the “acceptable” usability of the app cannot be generalized. The Thinking Aloud results revealed similar findings in terms of the wide range of time taken for each task. There may be different factors, such as education level, health literacy, and technical affinity that influence the perceived usability [51–54]. However, these factors could not be investigated as potential influencing factors in this study. To perform such subgroup analyses and to identify potential influencing factors, a larger sample is needed in future studies [55]. Similarly, the results of the pilot study by Bossen et al. showed a mean score of 73 points (SD 15) on the SUS [20]. In this Dutch study, participants mentioned the rigid and inflexible nature of the previous *Join2Move* website [20]. We have therefore developed an app that is inherently more intuitive and user friendly and adapted the *Join2Move* intervention to the German context. Nevertheless, the mean SUS score in our study decreased compared to the score of the Dutch *Join2Move* website. This could be explained by the fact that usability expectations are generally higher for an app, which by its nature should be more intuitive and flexible. In general, the *Join2Move* intervention was co-designed and developed with end-users directly from the beginning (as a website in the Netherlands) and continued in Germany, as it is crucial to involve end-users in the process of identifying system and usability problems [20, 56]. We therefore tried to incorporate the feedback from participants into the app. For example, we solved the usability issue of confusing translations in the information module. Due to resource and time constraints, not all of the usability issues identified during the development process could be addressed within the study period, such as the possibility of choosing more than one physical activity at a time or the integration of activity tracking tools or features such as saving favorite exercise. Therefore, it seems to be important to have clear agreements with the developing company and usability standards from the beginning, which need to be achieved in time and within the budget. Given the wide range of perceived usability of the app, a solution for further studies might be to conduct a larger app pretest. This approach would involve recruiting a heterogeneous sample of participants with, for example, different levels of education, technical skills, and levels of digital and health literacy [56].

In terms of effectiveness, this pilot study showed that the *Join2Move* program has potential for everyday practice in Germany. Even though the secondary outcomes did not significantly improve, trends favoring the intervention group were observed [18]. In contrast, we found a significant reduction in muscle strength (total work) measured by the isokinetic strength measurement. In general, the values of the isokinetic measurements decreased from baseline to follow-up. This might be because some of the participants also mentioned that they had a short-term increase in pain after performing the isokinetic measurement at t0; therefore, they might have been more cautious at the follow-up measurement. The influence of pain at different velocities within isokinetic measurements was also reported in other studies [57]. In addition, the physical activity level of the control group increased significantly more than that of the intervention group. This could be because they were randomized to the control group; however, they were still participating in a trial and were motivated to become active. Nevertheless, the intervention group also exhibited increased physical activity levels. Therefore, as in other studies, these findings indicate the potential of a stand-alone app as a treatment modality for patients with OA [55, 58–60].

Furthermore, we hypothesized that the intervention would be particularly suitable for early-stage OA in the context of the stepped care model [10]. Due to difficulties in recruiting only participants with early OA, we cannot test this hypothesis. However, the results suggest that a stand-alone app could be used at various stages of the patient journey, as indicated by our heterogeneous population sample (duration of OA complaints ranged from < 1 year to > 10 years). For example, if a patient has already received in-person therapy and an app-based intervention and is now experiencing pain and reduced physical functioning, this would be an ideal time to reintroduce exercise and physical activity using a stand-alone app as an intervention [60]. To further increase the effectiveness of the app-based program, it might be necessary to tailor the app to different stages of OA so that specific modules or levels can be selected depending on the patient’s current stage [60].

Further implications for improving the app could include examining other usability aspects or more innovative ideas, such as the integration of new features, social engagement, awards, more flexibility in the exercise program, the integration of an activity tracker, and more focus on behavior change techniques. A next step would be to explore patient needs and preferences for using a stand-alone app, similar to the findings of the Delphi study on patient and physiotherapist needs and preferences for a blended intervention [61], since not everyone might use an app at all [60, 62].

### Strengths and limitations

This study has several important strengths. The sample size achieved is sufficient for a pilot study, and the use of an online questionnaire, which was sent halfway through the study, kept the dropout rate relatively low. In addition, we translated and adapted an existing website that had been evaluated previously [19, 20]. The content was evidence-based, and important features such as goal setting and graded activities were already integrated. Furthermore, the assessors were blinded to group allocation.

Our study has several limitations. Blinding of participants was not possible due to the nature of the intervention. The sample size makes the calculation of effect sizes questionable, and the results should be interpreted with caution. Nevertheless, the study findings show positive trends and thus highlight the potential of stand-alone applications. Unfortunately, app usage data were not stored correctly for the entire study period and were not identifiable for all participants. Therefore, we were not able to use actual usage data; however, we were able to use questionnaire data related to the usage of the app. Future studies should pay attention to pretest the storage of usage data before initiation of the study.

## Conclusions

In summary, this pilot RCT showed that patients were satisfied and that the app was usable, demonstrating the potential of an app-based intervention. This study further supported the hypothesis that patients with hip and knee OA can benefit from a 12-week app-based physical activity and education program. Next, there was a significant and clinically relevant reduction in pain and an improvement in functioning in the intervention group. Thus, the use of an app in short-term management and treatment or as an app-based refresher for OA patients can be a valuable and promising tool for future OA care. There appears to be room for improvement in identifying patients for whom the app is suitable and for the right time to use a stand-alone app for patients with hip and/or knee OA.

### Supplementary Information

**Additional file 1.** *Join2Move* questionnaire (Questionnaire on the usability of the *Join2Move* app). The *Join2Move *questionnaire is a self-developed instrument for determining subjective usage time, user satisfaction and usability of the *Join2Move* app and its individual modules. In addition, information on any symptoms and adverse events that may have occurred was collected.

**Additional file 2.** *Join2Move* exercise program over twelve weeks with six different modules. The *Join2Move* exercise program is based on the *NEuroMuscular* *EXercise* (*NEMEX*) program and focuses on four domains: core stability/postural function, postural orientation, lower extremity muscle strength and functional exercises.

## Data Availability

The datasets used and/or analyzed during the current study are available from the corresponding author upon reasonable request.

## Abbreviations

CONSORT: Consolidated Standards of Reporting Trials
HOOS: Hip Disability and Osteoarthritis Outcome Score
ITT: Intention-to-treat
KOOS: Knee Injury and Osteoarthritis Outcome Score
OA: Osteoarthritis
RCT: Randomized controlled trial
