# Supplementary material for: Usability and preliminary effectiveness of an app-based physical activity and education program for people with hip or knee osteoarthritis – a pilot randomized controlled trial
Source: Arthritis Res Ther. 2024 Apr 10;26:83. doi: 10.1186/s13075-024-03291-z (PMC11005282; doi:10.1186/s13075-024-03291-z)
Supplement: Supplementary file 1 — Additional file 1. Join2Move questionnaire (Questionnaire on the usability of the Join2Move app). The Join2Move questionnaire is a self-developed instrument for determining subjective usage time, user satisfaction and usability of the Join2Move app and its individual modules. In addition, information on any symptoms and adverse events that may have occurred was collected. [file 13075_2024_3291_MOESM1_ESM.pdf]

Additional file 1

***Join2Move* questionnaire**

Questionnaire on the usability of the *Join2Move* app

This questionnaire refers to the period after your baseline measurement.

1. Module: Osteoarthritis-related complaints and treatments

1.1 Did any new osteoarthritis-related complaints occur in the weeks following your baseline measurement?

Yes	No
☐	☐

If so, which type of complaints?

- ☐ Pain in the affected hip or knee joint
- ☐ Swelling in the affected hip or knee joint
- ☐ Limited movement in the affected hip or knee joint
- ☐ Other: ______________________

1.2 On a scale from 0 (no symptoms) to 10 (greatest symptoms), how severe would you describe your current osteoarthritis-related symptoms? *(Please tick the appropriate number)*

0 1 2 3 4 5 6 7 8 9 10

1.3 Have you started any physiotherapy or medical treatment (e.g. acupuncture, injection of hyaluronic acid) in the weeks following your baseline measurement? (Multiple answers possible, unless "None" is ticked)

Physiotherapy	Medical therapy	None
☐	☐	☐

If yes, for which reason?

____

____

____

2. Adverse events

2.1 Were there any adverse events concerning your health in the weeks following your baseline measurement (e.g. falls, other accidents, complications such as extreme swelling or inflammation)?

Yes	No
☐	☐

If yes, which?

____

____

2.2 Have you increased your level of physical activity in the weeks following your baseline measurement?

Yes	No
☐	☐

If yes, to what extent?

approx. _____________ minutes per week

3. App usage

3.1 How often did you use the app in the weeks following your baseline measurement?

≥ 3 times per week	< 3 times per week	Not at all
☐	☐	☐

If „not at all“, for what reasons?

____

____

4. Module: exercises

4.1 How regularly did you perform the exercises provided by the app?

≥ 3 times per week	< 3 times per week	Not at all
☐	☐	☐

If „not at all“, for what reasons?

____

____

4.2 How difficult/easy was it to find the exercise module?

very difficult	difficult	easy	very easy
☐	☐	☐	☐

Comments:

____
____
____

4.3 How would you rate the usefulness/value of the exercises?

not valuable at all	not valuable	valuable	very valuable
☐	☐	☐	☐

Comments:

____
____
____

5. Module: physical activity

5.1 How regularly did you do the physical activity selected in the app (e.g. walking, cycling, swimming ...)?

≥ 3 times per week	< 3 times per week	Not at all
☐	☐	☐

If „not at all“, for what reasons?

____

____

5.2 How difficult/easy was it to find the module to promote your physical activity?

very difficult	difficult	easy	very easy
☐	☐	☐	☐

Comments:

____
____
____

5.2 How would you rate the usefulness/value of the physical activity module?

not valuable at all	not valuable	valuable	very valuable
☐	☐	☐	☐

Comments:

____
____
____

6. Module: Education

6.1 How regularly did you use the information material provided by the app (e.g. videos)?

≥ 3 times per week	< 3 times per week	Not at all
☐	☐	☐

If „not at all“, for what reasons?

____

____

6.2 How difficult/easy was it to find the education module?

very difficult	difficult	easy	very easy
☐	☐	☐	☐

Comments:

____
____
____

6.3 How would you rate the usefulness/value of the education module?

not valuable at all	not valuable	valuable	very valuable
☐	☐	☐	☐

Comments:

____
____
____

7. Usability and satisfaction with the *Join2Move* app

7.1 How would you rate the overall usability of the app? (*Please tick the appropriate box*)

0 1 2 3 4 5 6 7 8 9 10

7.2 **Overall, how satisfied are you with the app program so far? (Please tick the appropriate box)**

0 1 2 3 4 5 6 7 8 9 10
