# Supplementary material for: Usability and preliminary effectiveness of an app-based physical activity and education program for people with hip or knee osteoarthritis – a pilot randomized controlled trial
Source: Arthritis Res Ther. 2024 Apr 10;26:83. doi: 10.1186/s13075-024-03291-z (PMC11005282; doi:10.1186/s13075-024-03291-z)
Supplement: Supplementary file 2 — Additional file 2. Join2Move exercise program over twelve weeks with six different modules. The Join2Move exercise program is based on the NEuroMuscular EXercise (NEMEX) program and focuses on four domains: core stability/postural function, postural orientation, lower extremity muscle strength and functional exercises. [file 13075_2024_3291_MOESM2_ESM.pdf]

Additional file 2

***Join2Move* exercise program over twelve weeks with six different modules, respectively.**

Table 1 The Join2Move exercise program based on the NEuroMuscular EXercise *(NEMEX) program, which focuses on four domains: core stability/postural function, postural orientation, lower extremity muscle strength and functional exercises*

Modules	Module 1	Module 2	Module 3	Module 4	Module 5	Module 6
**Week**	**Week 1,2**	**Week 3,4**	**Week 5,6**	**Week 7,8**	**Week 9,10**	**Week 11,12**
**Exercise**	Exercise 1	Exercise 4	Exercise 7	Exercise 10	Exercise 13	Exercise 16
**Name**	Knee extension	Active knee extension	Knee extension while sitting	Knee extension with an elastic band	Knee flexion with resistance	Lunge (activation of the popliteus muscle)
**Picture**	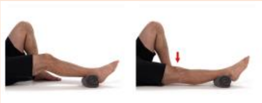	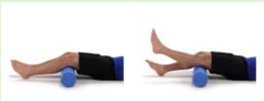	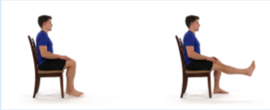	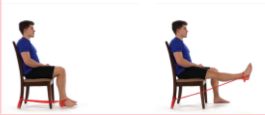	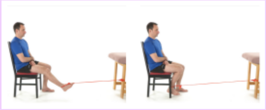	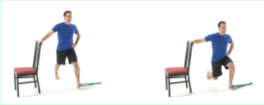
**Exercise**	Exercise 2	Exercise 5	Exercise 8	Exercise 11	Exercise 14	Exercise 17
**Name**	Hip abduction while standing	Half squat	Sit down and stand up	Ascending/ descending	Sliding hip extension	Eccentric plantar flexion
**Picture**	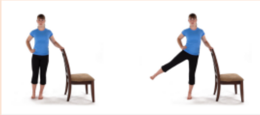	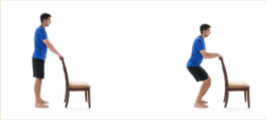	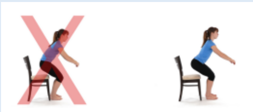	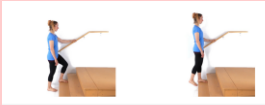	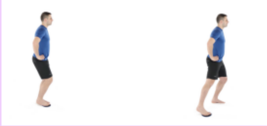	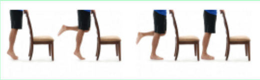
**Exercise**	Exercise 3	Exercise 6	Exercise 9	Exercise 12	Exercise 13	Exercise 18
**Name**	Pelvic lift	Activating transverse abdominal muscles	FABER-stretching	Stretching the gluteal muscles (gluteus maximus)	Standing quadriceps stretch	Standing on one leg (eyes closed)
**Picture**	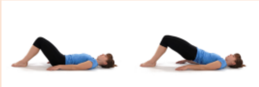	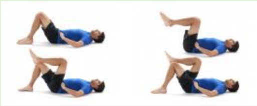	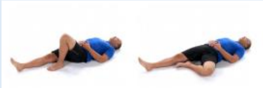	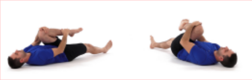	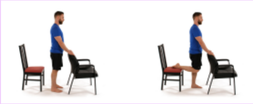	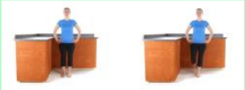
